# Tracking induced forgetting across both strong and weak memory representations to test competing theories of forgetting

**DOI:** 10.1038/s41598-021-02347-3

**Published:** 2021-11-29

**Authors:** Ashleigh M. Maxcey, Zara Joykutty, Emma Megla

**Affiliations:** 1 Department of Psychology, Vanderbilt University, Wilson Hall, 111 21st Ave S, Nashville, TN 37212 USA; 2 Department of Psychology, University of Chicago, Chicago, IL USA

**Keywords:** Psychology, Human behaviour

## Abstract

Here we employ a novel analysis to address the question: *what causes induced forgetting of pictures?* We use baseline memorability as a measure of initial memory strength to ask whether induced forgetting is due to (1) recognition practice damaging the association between the memory representation and the category cue used to activate the representation, (2) the updating of a memory trace by incorporating information about a memory probe presented during recognition practice to the stored trace, (3) inhibitory mechanisms used to resolve the conflict created when correctly selecting the practiced item activates competing exemplars, (4) a global matching model in which repeating some items will hurt memory for other items, or (5) falling into the zone of destruction, where a moderate amount of activation leads to the highest degree of forgetting. None of the accounts of forgetting tested here can *comprehensively* account for both the novel analyses reported here and previous data using the induced forgetting paradigm. We discuss aspects of forgetting theories that are consistent with the novel analyses and existing data, a potential solution for existing models, proposals for future directions, and considerations when incorporating memorability into models of memory.

## Introduction

Forgetting can occur volitionally, such as intentionally avoiding unwanted memories, or incidentally, such as the inability to recall the name of a restaurant. In the case of incidental forgetting, it has been shown that reactivating a memory representation (e.g., *Etch*, the name of one Nashville restaurant) can lead to the forgetting of related memories (e.g., *Husk*, the name of another Nashville restaurant). Forgetting as a consequence of accessing related information occurs following the retrieval of words (i.e., retrieval-induced forgetting^1^) and recognition of pictures (i.e., recognition-induced forgetting). Several theories of forgetting may account for induced forgetting. Here we implement a novel analysis to test the ability of leading theories to account for induced forgetting^2^.

In a typical recognition-induced forgetting task^3–6^, participants are instructed to study sequentially presented images. The studied images include different exemplars from several categories (e.g., six lamps, six vases, six chairs, six mugs). Participants’ memory is then tested for a portion of the exemplars from a subset of the categories (e.g., three lamps, three vases) in the practice phase. This design creates three different object types. *Practiced* objects are the objects that subjects practice recognizing in the second phase. *Related* objects belong to practiced categories and are the remaining half of the category that is not practiced. *Baseline* objects belong to the categories of objects that are not practiced. Finally, in the third phase, memory for all studied images is tested. Performance in this test phase shows the hallmark of induced forgetting, worse memory for related objects relative to baseline objects. This difference in memory for related and baseline objects is remarkable because both related and baseline objects were only seen once, during the initial study phase. Nevertheless, related objects are consistently forgotten following recognition practice.

Recognition-induced forgetting affects memory for a variety of memoranda, including pictures of everyday objects^6^, faces^4^, words^5^, and emotionally arousing images^7^. Induced forgetting occurs following recognition and rejection^8^, even when the viewer has knowledge of the forgetting effect and is instructed to resist forgetting^3^. This incidental forgetting appears more robust than intentional forgetting^9^. Despite the robustness of this effect, the mechanism underlying induced forgetting remains unclear (but see^10^ for a proposed process model).

### Memorability

Induced forgetting is measured in relative memory performance (i.e., measured in hit rate and d′) averaged across object type (i.e., practiced, baseline, related), where memory for related items is reliably worse than memory for baseline items. Here we further subdivide changes in relative memory strength across object type by memorability. Images have an intrinsic property, known as *memorability*, that influences the likelihood that the image will later be remembered or forgotten across observers^11^. This means that memorability can be quantified for individual images^12^. Image memorability is calculated by averaging the overall memory performance for an image across observers during a recognition task, resulting in a memorability scale ranging from 0 (images everyone forgets) to 1 (images everyone remembers)^13,14^.

Memorability reflects changes in activation strength because varying levels of memorability are tracked across patterns of activation in inferotemporal cortex^15^, as well as neural pattern differences in medial temporal lobe and memory areas^16^, and semantic areas like the anterior temporal lobe^17^. Memorability is consistent across various temporal delays, from 36 s^18^ to 1 week^19^. Additionally, memorability can be measured in one experiment and then applied in other experimental paradigms^20^ and generalizes between subjects^17^. Although extrinsic contextual effects can modulate intrinsic memorability^21^, memorability scores are reliable and predictive^22^ when images are presented in a random sequence. We use this randomized approach in recognition-induced forgetting. This allows us to use baseline memory performance in induced forgetting studies (i.e., accuracy in the final memory test phase for object categories that were studied once and not involved in the practice phase) as a measure of image memorability. We can use this baseline performance to sort objects into low, medium, and high memorability categories. From there, we can see how recognition impacts memories when their initial memory strength is low, medium, or high. Here we use this method to ask whether induced forgetting is due to (1) damaged associations between cue and trace, (2) trace updating, (3) inhibition, (4) global matching models, or (5) the zone of destruction.

## Method

### Induced forgetting experiment

**Figure 1:**
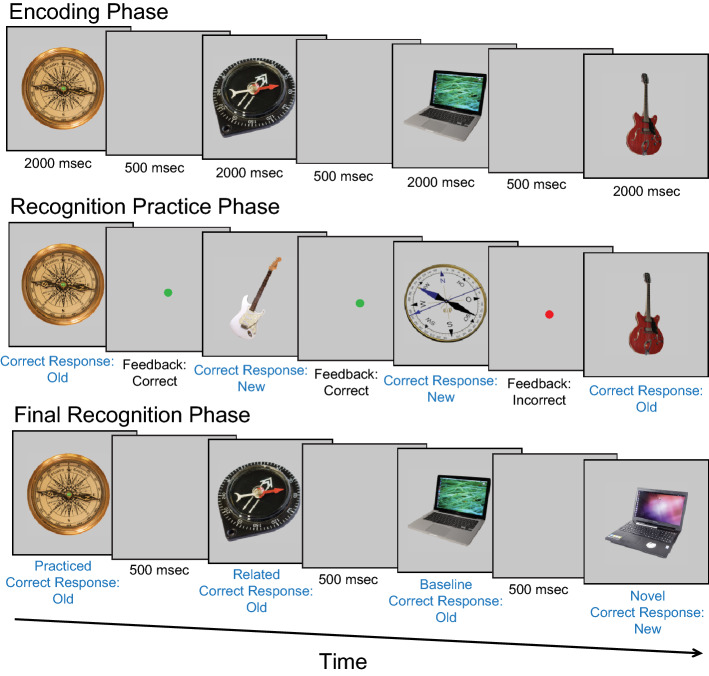
Brief overview of Fukuda, Pall^8^ methods. Objects were sequentially presented for 2 s each during the encoding phase. Then memory for half of the objects from half of the categories was tested in the recognition practice phase. Memory for all objects was then tested in the final recognition phase.

Here we report a novel reanalysis of published data to calculate memorability scores, a method done previously^23^, which we used to test various theoretical accounts of induced forgetting. Refer to Experiment 1 of Fukuda, Pall^8^ for a more detailed overview of the experimental methods that produced the data we analyze here. In brief, the original study aimed to determine whether both recognition of old objects and rejection of new objects was required to induce the forgetting of related objects. The authors found that recognition and rejection independently could induce forgetting with reliably worse memory for related compared to baseline objects. We selected this induced forgetting dataset to analyze for memorability due to the large number of subjects who participated in the study and its diverse stimulus set. The original experiment started with an encoding phase in which subjects were sequentially shown objects to remember (see Fig. 1 for a general overview of the methods). For each subject, there was a total of 180 objects (drawn from a larger stimulus set) presented during this phase, each shown twice, for a total of 360 trials. Each object was presented in random order for 2 s with 500 ms between images.

The encoding phase was followed by a recognition practice phase in which objects from a portion of the studied object categories (60/90) were presented sequentially. Subjects were asked to respond whether the presented object was *old* or *new* in addition to their confidence level (60%, 80%, or 100% confident). Three conditions were interleaved during this phase; one third of the practiced object categories only presented *old* objects (*all-old* condition), another third only presented *new* objects (*all-new* condition), and the final third of object categories presented the typical 50/50 *old/new* divide of objects (*mixed* condition). This within-subject practice phase manipulation was the critical manipulation to determine whether both recognition of old objects and rejection of new objects was required for induced forgetting. There was a total of 240 trials in the recognition practice phase, and the color of the fixation dot changed upon response to provide subjects with 100% valid feedback (green if correct, red if incorrect) for the trial.

Recall that the practice phase of induced-forgetting studies creates three different object types. Old objects presented during the practice phase are called *practiced* objects. Old objects in the same categories as these practiced objects, but that were not shown in the practice phase, are called *related* objects. Old objects belonging to categories not presented at all during the practice phase are called *baseline* objects.

In the final recognition phase, subjects completed a similar old/new recognition task as the recognition practice phase, but no feedback was provided. From the object categories in the all-old and mixed conditions, 20 practiced, 20 related, and 20 baseline objects were presented as old objects during the final phase. As no old objects were presented during the practice phase in the all-new condition, 40 related objects and 20 baseline objects were presented from these categories during test. Ninety new objects (one from each object category) were also presented during this phase for a total of 270 trials. The full object set drawn from across subjects included 304 images from 48 different categories, with 5–7 exemplars from each category.

### Memorability score analysis

First, we collapsed across confidence ratings and practice conditions (i.e., all-new, all-old, and mixed conditions) as the magnitude of induced forgetting was found to be consistent across these in the original experiment^8^. Next, we calculated the memorability score of each image used in^8^ by averaging together all hit rates (H) and false alarm rates (F) to that particular image. As related and practiced objects experienced induced forgetting and practiced effects during the experiment, we only factored hit rates and false alarm rates for when the image was used as a baseline object into the averages. We used these averaged rates to calculate the image’s d′ [or memorability score, calculated as z(H) − z(F)]. To avoid infinite values when calculating d′, we adjusted hit rates and false alarm rates of 1.0 or 0 to 1 − (1*/*[*2*N]) and 1/(*2N*), respectively, where N is the number of subjects for that given calculation and image^24^.

We then divided the d′ scores of baseline objects into roughly equal bins representing low, medium, and high memorability. Any objects with a baseline d′ score of zero or below were discarded. Additionally, any objects with less than three subjects contributing to the memorability score were also discarded. The purpose of excluding any objects with less than three subjects contributing to the memorability score was to reduce noise. As object categories themselves have been shown to have their own intrinsic memorability values^25^, we excluded any object categories that did not contribute at least one object to each of the three memorability bins (i.e., low, medium, and high). This means that there were no object categories that were only lowly or highly memorable in our final analysis. After excluding these categories, there was an average of 101.33 (range = 98–104) objects remaining in each resulting memorability condition, with an average of 9 subjects contributing to the memorability score for each object across conditions.

To confirm that memory performance was generally consistent across participants, we used the established split-half rank correlation analysis on the calculated memorability scores^13,14^. Specifically, we calculated two separate memorability scores (d′) for each object across two random halves of subjects. We then performed a Spearman rank correlation on these two halves to determine the consistency of the memorability ranking of each image. We performed this analysis 1000 times, resulting in an average correlation coefficient value across these iterations. If the memorability scores are consistent, we should find a positive correlation across the two groups, meaning that participants remember and forget the same items. Indeed, we found memorability scores reliably correlate with an average p-value of p = 0.0101. Data and analyses can be found on Open Science Framework (https://osf.io/87e26/).

Next, we confirmed that these memorability bins were not contaminated by an uncontrolled aspect of the original experiment. To this end, we ran an additional experiment to confirm that memory for low-memorability items was indeed lower than medium-memorability items, and that memory for high-memorability items was indeed higher than medium-memorability items. All methods were carried out in accordance with the relevant guidelines and regulations: informed consent was obtained from all subjects and all procedures were approved by the Vanderbilt University Institutional Review Board. In this experiment, subjects were shown two randomly selected objects from each object category presented in the original experiment for 5 s each and instructed to remember them for a later memory test. Then subjects' memory was tested for the 96 studied objects, using another 96 images from the stimulus set as test lures. Across 56 subjects we confirmed that memory for objects in the low-memorability bin (0.7516) is reliably lower than memory for objects in the medium-memorability bin (0.7722, t(55) = 2.021, p = 0.048, d = 0.270) and memory for objects in the high-memorability bin was higher than memory in the medium-memorability bin (0.7948, t(55) = 2.396, p = 0.020, d = 0.320).

**Figure 2:**
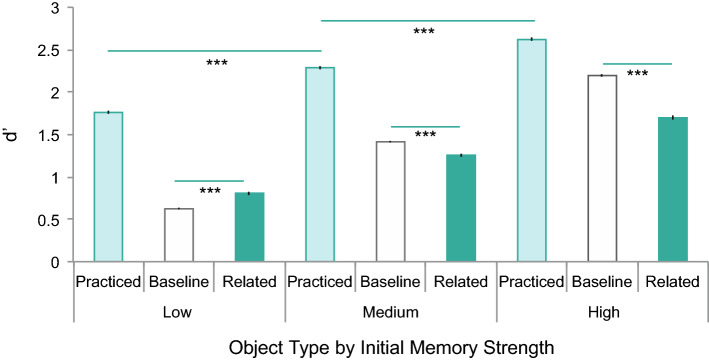
Fukuda, Pall^8^ d′ data sorted by object type across low-, medium-, and high-strength conditions. Initial memory strength categories were determined using baseline performance (white bars). Error bars represent standard error. Induced forgetting is reliable in the medium- and high-strength conditions, with the magnitude of induced forgetting larger in the high-strength condition. The benefit of practice reliably increases across all three conditions. Rather than practice inducing forgetting in the low-strength condition, practice had the opposite effect, reliably increasing memory for related objects.

Having established whether each individual picture belonged in the low, medium, or high memorability bin, we next sorted objects within each bin by object type. The result of this sorting is shown in Fig. 2. In the experiment we analyze here, object type was randomly determined across participants such that each object was used as a practiced, baseline, and related object between subjects. We confirmed that these baseline memorability scores were accurate by confirming that the same staircase trend shown for baseline objects (Fig. 2, white bars), of d′ increasing across low, medium, and high memorability scores (F(2,301) = 833.2, p < 0.001, η^2^_p_ = 0.847), replicated across practiced (F(2,301) = 53.92, p < 0.001, η^2^_p_ = 0.264) and related (F(2,301) = 45.90, p < 0.001, η^2^_p_ = 0.234) objects. Further, subjects were better at remembering the related, highly memorable items (the forgotten items following induced forgetting) relative to the less memorable baseline items from the medium (t(202) = 3.606, p < 0.001, d = 0.505) and low (t(196) = 16.01, p < 0.001, d = 2.276) memorability categories. These analyses confirm that our memorability scores are appropriate measures of initial memory strength increasing from low to high across the three memorability bins.

Given that we used memorability scores as a proxy for initial memory strength to test competing theories of memory that use the term *memory strength*, from here on we use *initial memory strength* (or *low-, medium-,* and *high-strength*) to refer to the calculated memorability scores. Next, we consider potential theoretical explanations of forgetting that can be tested with this novel analysis.

## Is induced forgetting due to damaged associations between the category cue and the related items?

If a memory representation is disconnected from its category cue used to activate the representation, it should be nearly impossible to conjure up the memory^26^. Consequently, if recognition practice damages the association between the category cue and the related objects, then memory for related items should never be raised following recognition practice as there would be no way to bring the related item memory to mind. Here we capitalized on the memory strength analysis described above to determine whether memory for related items is ever raised above baseline. The comparison against baseline is ideal because, unlike related items, no members of baseline categories were practiced that could weaken the category cue to these items. Therefore, the cue associated with baseline items would be unaffected by recognition practice. We found that recognition practice of low-strength items actually *improves* memory for related items, bolstering memory reliably above baseline (t(99) = − 4.426, p < 0.001, d = − 0.443). The absence of induced forgetting for low-strength items shows that recognition practice does not damage the association between related items and their associated category cues, ruling out this explanation of forgetting, at least for low-strength items.

The enhancement of related items relative to baseline is consistent with the general activation of all items in the category in comparison against the memory probe. One reason the related items may not be suppressed relative to baseline is that the enhancement is not followed by the need to suppress these non-target members of the category, because their memory strength is too low to cause competition^27^.

**Figure 3:**
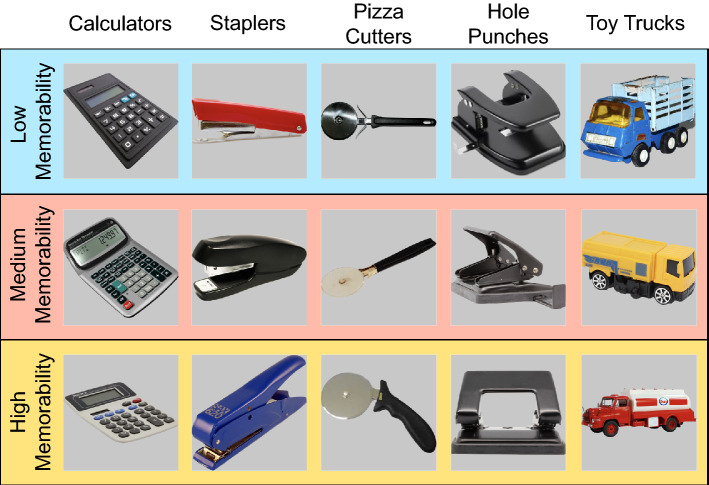
Example of stimulus categories with exemplars included in low-, medium-, and high-memorability conditions. These examples demonstrate that the three memorability conditions are not grouped based on obvious perceptual differences.

One may argue that perhaps forgettable objects are simply worse (i.e., less representative) members of their category relative to memorable objects. On the contrary, memorability does not reflect an object’s typicality within a category^28^. In Fig. 3 we show an example of the images in each category in the present study, demonstrating that memorability differences within a category are not due to obvious perceptual differences.

## Is induced forgetting due to trace updating?

According to trace updating (also known as *differentiation*), when a memory probe is presented, memory traces (i.e., representations) similar to the memory probe are activated and potentially updated^29^. Activation depends on the similarity between features of the memory probe and features stored of the memory trace. When the trace is strong enough and similar enough to the memory probe, this prompts the memory system to determine a match has been found (this process can be modeled mathematically, without the assumption that the participant overtly believes there is a match), triggering it to incorporate information about the probe to the stored trace. Unlike models that assume each opportunity to study an item results in separate noisy traces^30–33^, trace updating assumes that the same memory trace is updated, with information added upon each study opportunity, preventing the trace from interfering with weaker representations^34,35^. This process of trace updating only occurs when the memory system determines it recognizes the item. If the item is not recognized, a new trace is created. Trace updating occurs in *error* when the trace is not a match, but was close enough to the probe that the subject believed it was a match and trace updating was triggered. In this case, it makes the trace *less* similar to the original item that produced the trace because the features added are from a different item, the test probe. In the case of induced forgetting, related items are from the same categories as practiced items and are therefore likely to be similar enough to the memory probe to be activated during the practice phase. This may result in the memory traces of related items being updated in error. This could explain why at test when the related item is presented as a memory probe, it is not correctly identified and is deemed forgotten.

If trace updating explains induced forgetting, then we should see induced forgetting only occurring for medium-strength items and not for low- or high-strength items. Specifically, if initial memory strength for a related item is low enough, then its trace should not be activated during practice, or if activated will not match well enough to prompt a decision about the trace matching the current test probe. This failure to match means trace updating would not occur, and the unaltered memory trace of the related item would still closely resemble its memory probe. If the trace of the low-strength related item maintains its original fidelity because it was not updated, then subjects will correctly respond that the related item is old and forgetting will not occur. At the other extreme, if initial memory strength is high for a related item, then an object will efficiently be correctly identified as different from the memory probe and trace updating will also not occur. Again, if the trace of the high-strength related item maintains its original fidelity because it was not updated, then subjects will correctly respond that the related item is old and forgetting will not occur. Therefore, if trace updating underlies forgetting, then forgetting should not occur for low- or high-strength items. Here we found that while forgetting does not occur for low-strength items, it does occur for high-strength items (t(99) = 8.924, p < 0.001, d = 0.901). The presence of induced forgetting for high-strength items is inconsistent with a trace updating explanation of induced forgetting, at least for high-strength items.

An additional test of trace updating would be to examine performance from the practice phase across the three memory strength bins. According to trace updating, subjects who recognize 100% of old objects would update all practiced traces, regardless of the object’s initial memory strength, while subjects who recognize 0% of old objects would have no updated traces, regardless of initial memory strength. The data we analyze here has three different conditions in the practice phase (described above in the Methods section), and was not run balancing initial memory strength, rendering this proposed analysis underpowered here.

## Is induced forgetting due to inhibition?

One popular explanation of induced forgetting is the inhibition account^36^. The inhibition account argues that induced forgetting occurs as a consequence of the activation of related items during the practice phase. Specifically, the presentation of any item from the same object category leads to the activation of all same category items, creating conflict in effectively selecting the practiced item. Resolving this conflict to identify the practiced item requires the inhibition of related items, a suppression that endures into the test phase and is measured as forgetting.

One prediction of the inhibition account is interference dependence. Interference dependence states that the degree of forgetting depends on the degree of interference created by related items. In other words, related items with stronger memory traces create more interference when activated during practice, thereby requiring more inhibition to suppress^36^. Here we can test this prediction that forgetting varies as a function of interference from related items using initial memory strength to quantify the degree of interference from a related item. Interference dependence predicts that the stronger the interference—here measured by initial memory strength of related items—the stronger the degree of forgetting. Consistent with this prediction, the magnitude of induced forgetting is larger for high-strength items relative to medium-strength items (t(202) = 4.785, p < 0.001, d = 0.671).

A second prediction of the inhibition account, retrieval specificity, states that forgetting should only occur following tasks requiring active retrieval, where participants are explicitly prompted to retrieve previous memory representations^36^. However, several studies have shown that induced forgetting still occurs when the old-new recognition judgment task in the practice phase is replaced with a simple restudy task^5,37^, inconsistent with this prediction. One may argue that the degree in which restudy involves implicit, active recognition is unclear. Although participants are not explicitly asked to retrieve memory representations, it still may be an automatic response to viewing an image. Therefore, these studies alone do not provide definitive evidence against the inhibition account (but see^38^). Taken together, we have presented evidence that can be interpreted both for and against the inhibition account. Is there an explanation of forgetting that is consistent with all previous induced forgetting data and the present novel analyses?

## Does a global matching model explain induced forgetting?

Global matching models explain memory retrieval using a global matching mechanism that determines the similarity between a memory probe and each representation stored in memory^39^. The similarity values are then combined to calculate a global similarity index, reflecting the similarity between the test probe and items in memory, used to support a recognition response. The specific calculations depend on the particular global matching model (for an excellent introduction to these models see^39^).

Global matching models can account for worsening performance when a list gets longer (i.e., list-length effects) because each added item to the list contributes additional variance to the global similarity index, increasing variance in the distributions representing targets and lures, and decreasing the signal-to-noise ratio. Following this same logic, a number of global matching models would predict induced forgetting^40–42^ because, in the same way *additional* items hurt memory (i.e., the list-length effect, described above), *repeating* some items hurts memory for other items^43,44^. This is because when the memory for an item is strengthened by increased exposure (e.g., through study or repetition)^39^, memory retrieval for other items will be impaired because each repetition adds to the contents of memory (similar to new items impairing memory), increasing variance.

Within these features of global matching models, *testing* also adds strength to the traces of studied items because when global similarity is computed at retrieval, strong memories beat weak memories. This means that both recognition practice (i.e., testing) and restudy (i.e., repetition and additional exposure time) in the induced forgetting paradigm would increase the overall storage strength of the trace. Thus global matching models can explain why memory for practiced items is higher, even following restudy^5^, a finding not easily accounted for by an inhibition model^36^. Similarly, global matching models can also account for memory of practiced objects increasing across all levels of memory strength (shown in Fig. 2, F(2,301) = 53.92, p < 0.001, η^2^_p_ = 0.264, each individual comparison p < 0.001), because the initial memory strength increases from low-strength to high-strength categories.

Some global matching models incorporate non-linearity into the global similarity computation (i.e., traces are not all weighted equally), which can result in differential rates of forgetting across similarity values, such as producing larger changes among neighboring representations the closer they are to the test probe^39,42,45,46^. This non-linearity across the similarity function could account for differential rates of forgetting across various memory strengths. Here we show that the magnitude of induced forgetting is larger for high-strength items relative to medium-strength items (t(202) = 4.785, p < 0.001, d = 0.671). Although non-linearity in these models might account for changes in the magnitude of forgetting, these models would not predict the reliable *facilitation* effect between baseline and related objects in the low-strength condition (t(202) = 4.426, p < 0.001, d = 0.443, reliable in the opposite direction predicted by induced forgetting).

## Does the zone of destruction explain induced forgetting?

A theory of forgetting that is reminiscent of the story of Goldilocks and The Three Bears posits that weak and strong memories are not forgotten, but rather that moderately activated memories fall into the ‘zone of destruction’ and are forgotten^47–50^ even when the degree of interference within categories is accounted for^51^. The zone of destruction account posits that only medium- and high-strength memories are engaged in competition at retrieval, but the medium strength items are not sufficiently strong to win the competition and are thus forgotten. This account makes specific predictions across the three levels of initial memory strength here. If the medium strength items are indeed most susceptible to forgetting, then the magnitude of induced forgetting for the middle level of memory strength should be greater than the magnitude of induced forgetting operating over levels of low- and high-memory strengths. Contrary to this prediction, the magnitude of induced forgetting is larger for high-strength items relative to medium-strength items (t(202) = 4.785, p < 0.001, d = 0.671). Therefore, the zone of destruction account cannot explain forgetting in the medium- or high-strength conditions.

## Discussion

Here we have presented a novel analysis employing initial memory strength to watch induced forgetting unfold across weak and strong memories. We have described the results of these analyses, along with results from previous studies, from the viewpoint of potential theoretical accounts of induced forgetting. We have argued that these novel analyses, combined with existing data, cannot be *comprehensively* explained by (1) damaged associations between cue and trace, which cannot account for the low-strength condition, (2) trace updating, which cannot account for the high-strength condition, (3) inhibition, which cannot account for previously published results, (4) global matching, which cannot account for the low-strength condition, or (5) the zone of destruction, which cannot account for the medium- or high-strength conditions.

### Facilitation of weak representations

None of the proposed explanations appear to account for the *facilitation* of related items in low-strength conditions, apart from perhaps the inhibition account. From a process model standpoint, it is easy to see how exposure to items from a category (i.e., recognition practice or restudy in the second phase of induced forgetting experiments) may lead to activation of all members of the category, akin to spreading activation and a rising tide raising all boats. Due to their low-strength status, it may be that the subsequent suppression of low-strength items is not necessary because they cause little to no competition during retrieval. While the concept of forgetting induced only when necessary is predicted by several proposed accounts above, it is unclear why these accounts would predict the *facilitation* of related items found in the low strength category. Perhaps the inhibition account comes closest to an explanation, yet, as mentioned above, restudy induces forgetting^5,8^ contrary to the retrieval specificity prediction of the inhibition account. Crossing a memorability manipulation with the restudy condition of Maxcey, Janakiefski^5^ may provide a further compelling test of the inhibition account.

### Criterion shifts

If there are underlying mechanistic differences between different levels of memorability, then different theories of forgetting may account for forgetting of low-, medium-, and/or high-strength memories. Here we used evidence from any three levels of memorability to assess a theory of forgetting, yet it may be the case that different theories of forgetting can account for outcomes at different levels. As noted in our analyses above, one may argue that certain theories of forgetting can successfully account for specific levels of memorability.

On the other hand, it may be that an individual account of forgetting could be applied to all three levels of memorability by adding an assumption of criterion shifts. Specifically, perhaps different decision criteria are applied for stimuli of differing memory strengths. When collapsing across all objects, and thus all levels of memorability, criterion shifts cannot explain induced forgetting^37^. However, evidence that false alarm rates do not reliably differ between practiced and non-practiced items^2,37,52^ may reflect shifting criteria across memories of different strengths.

### Modeling memorability

It is perhaps unsurprising that none of the proposed explanations appear to account for the memorability analyses presented here. From a representational standpoint, it is unclear exactly what memorability represents and thus it may be difficult to incorporate into existing models. According to global matching models, a ‘memorable’ stimulus is less similar to other stimuli, rendering it less susceptible to interference^53^. However, if high memorability meant less similarity, one would predict less induced forgetting for high-strength items, which is opposite the pattern observed here. This is because there would be no need to induce forgetting, if one assumes a functional role of induced forgetting is to enable the selection of targeted memories.

Complicating matters further, global similarity is a changing variable that represents variance across trials for both targets and lures^39^ whereas memorability can be measured with one sample (e.g., online participants) and generalized to another sample (e.g., intracranial EEG epilepsy patients)^17^. In this way memorability also cannot be represented by a variable that incorporates encoding conditions, because variables at encoding differ across contexts and across samples, yet memorability stays the same. Instead, memorability may be more appropriately represented by self-similarity (e.g., associations between features of the object itself)^39^. When accounting for induced forgetting, future tests of leading memory models may incorporate these results by introducing a reliable and predictive representational variable for memorability.

## Data Availability

Data and analyses are located on Open Science Framework: https://osf.io/87e26/.
